# Evolution and Diversity of Clonal Bacteria: The Paradigm of *Mycobacterium tuberculosis*

**DOI:** 10.1371/journal.pone.0001538

**Published:** 2008-02-06

**Authors:** Tiago Dos Vultos, Olga Mestre, Jean Rauzier, Marcin Golec, Nalin Rastogi, Voahangy Rasolofo, Tone Tonjum, Christophe Sola, Ivan Matic, Brigitte Gicquel

**Affiliations:** 1 Unité de Génétique mycobactérienne, Institut Pasteur, Paris, France; 2 Unité de la Tuberculose et des Mycobactéries, Institut Pasteur de Guadeloupe, Abymes, Guadeloupe; 3 Unité de la Tuberculose et des Mycobactéries, Institut Pasteur de Madagascar, Antananarivo, Madagascar; 4 Centre for Molecular Biology and Neuroscience and Institute of Microbiology, University of Oslo, Oslo, Norway; 5 Centre for Molecular Biology and Neuroscience and Institute of Microbiology, Rikshospitalet, Oslo, Norway; 6 Institut National de la Santé et de la Recherche Médicale U571, Faculté de Médicine, Université Paris V, Paris, France; Centre for DNA Fingerprinting and Diagnostics, India

## Abstract

**Background:**

*Mycobacterium tuberculosis* complex species display relatively static genomes and 99.9% nucleotide sequence identity. Studying the evolutionary history of such monomorphic bacteria is a difficult and challenging task.

**Principal Findings:**

We found that single-nucleotide polymorphism (SNP) analysis of DNA repair, recombination and replication (3R) genes in a comprehensive selection of *M. tuberculosis* complex strains from across the world, yielded surprisingly high levels of polymorphisms as compared to house-keeping genes, making it possible to distinguish between 80% of clinical isolates analyzed in this study. Bioinformatics analysis suggests that a large number of these polymorphisms are potentially deleterious. Site frequency spectrum comparison of synonymous and non-synonymous variants and Ka/Ks ratio analysis suggest a general negative/purifying selection acting on these sets of genes that may lead to suboptimal 3R system activity. In turn, the relaxed fidelity of 3R genes may allow the occurrence of adaptive variants, some of which will survive. Furthermore, 3R-based phylogenetic trees are a new tool for distinguishing between *M. tuberculosis* complex strains.

**Conclusions/Significance:**

This situation, and the consequent lack of fidelity in genome maintenance, may serve as a starting point for the evolution of antibiotic resistance, fitness for survival and pathogenicity, possibly conferring a selective advantage in certain stressful situations. These findings suggest that 3R genes may play an important role in the evolution of highly clonal bacteria, such as *M. tuberculosis*. They also facilitate further epidemiological studies of these bacteria, through the development of high-resolution tools. With many more microbial genomes being sequenced, our results open the door to 3R gene-based studies of adaptation and evolution of other, highly clonal bacteria.

## Introduction

Despite their different tropisms, phenotypes and pathogenicities, *M. tuberculosis* complex (MTC) strains are highly clonal: their nucleotide sequences are 99.9% identical and 16S rRNA sequences do not differ between MTC members, with the exception of *M. canetti*. It is difficult to study the evolutionary history of such mono-morphic bacteria [1]. Several methods based on polymorphic loci or the sequencing of housekeeping genes have been used to distinguish between *M. tuberculosis* complex isolates [2]–[9]. However, they have provided only low resolution and sparse functional information on how strains evolve and adapt to changes in environmental selection pressures, such as immune pressures and antimicrobial drug treatment.

Allelic variations in bacteria arise from random mutation, which may or may not be subject to selective pressure, horizontal gene transfer or recombination events. Evidence for horizontal transfer and recombination has recently been obtained, but exchange of genetic material in *M. tuberculosis* seems only to have occurred in the distant past [10], [11]. Other mechanisms, such as DNA repair, recombination and replication (3R) may have driven more recent *M. tuberculosis* evolution. *M. tuberculosis* may be regarded as a possible natural mutator, as there are no genes for components of the DNA mismatch repair system in its genome. In addition, within the W-Beijing family of strains, characteristic variations have already been found in DNA repair genes, null alleles of which have been shown to lead to an increase in spontaneous mutation frequency in *M. smegmatis* [12], [13]. A previous analysis of the *M. tuberculosis* H37Rv genome identified homologs of genes involved in the reversal or repair of DNA damage in *E. coli* and related organisms [14]. We analyzed most of these homologs, comprising a comprehensive set of 56 3R system components

## Results and Discussion

### Polymorphisms in global MTC strains

**Figure 1:**
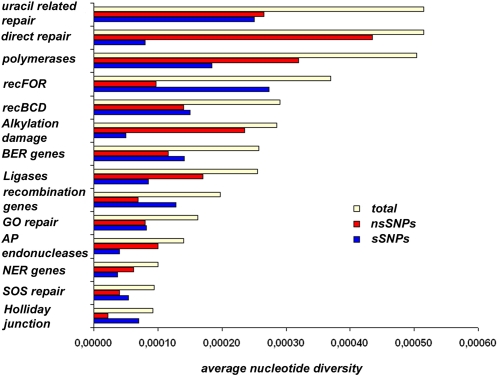
Average nucleotide diversity by gene class. It was calculated based on the results for the clinical strains according to the class of 3R genes analyzed. Holliday Junction resolving genes, 4407 nucleotides-4 genes. SOS repair, 16893 nucleotides-10 genes. NER genes, 18108 nucleotides-5 genes. AP endonucleases, 1635 nucleotides-2 genes. GO repair, 5850 nucleotides-8 genes. Recombination involved genes, 30567 nucleotides-18 genes. Ligases, 6957 nucleotides-2 genes. BER genes, 8328 nucleotides-10 genes. Alkylation damage, 3216 nucleotides-4 genes. RecBCD, 8307 nucleotides-3 genes. RecFOR, 2568 nucleotides-3 genes. Polymerases, 7857 nucleotides-5 genes. Direct repair, 1989 nucleotides-2 genes. Uracil related repair, 1149 nucleotides-2.

**Figure 2:**
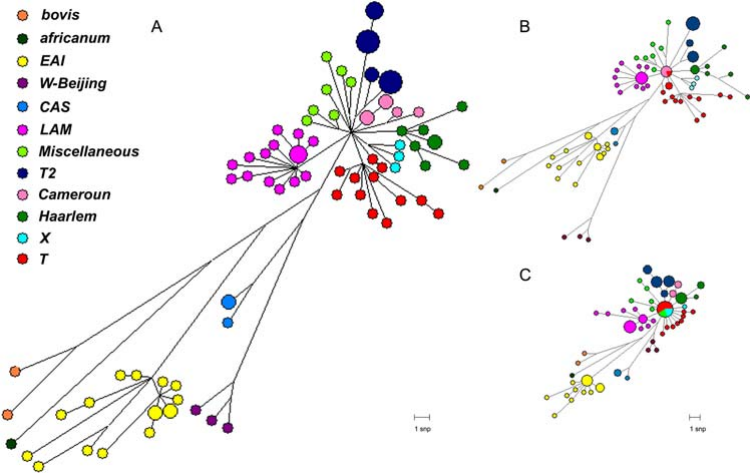
(A) Phylogenetic network based on the total set of SNPs. This phylogenetic network was constructed using the median-joining algorithm with a final set of 252 SNPs characterized in 92 clinical strains of the *Mycobacterium tuberculosis* complex (MTC). (B) Phylogenetic network based on the nsSNPs. This phylogenetic network was constructed using the median-joining algorithm with a final set of 163nsSNPs characterized in 92 clinical strains of the MTC. (C) Phylogenetic network based on the sSNPs. This phylogenetic network was constructed using the median-joining algorithm with a final set of 89 sSNPs characterized in 92 clinical strains of the MTC. Deletions were excluded from the analysis. Clinical isolates are classified with a color code, according to their spoligotype-based family. Node sizes indicate the number of strains belonging to the same haplotype.

**Figure 3:**
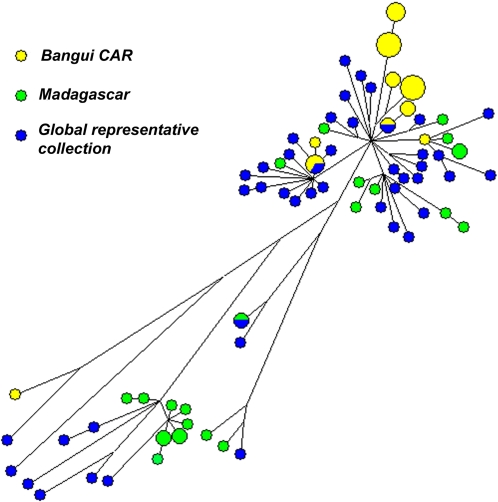
Geographic origin of the haplotypes identified. This phylogenetic network constructed using the median-joining algorithm with a final set of 252 SNPs characterized in 92 clinical strains of the *Mycobacterium tuberculosis* complex (MTC). Deletions were excluded from the analysis. Geographical origin is classified with a color code. Node sizes indicate the number of strains belonging to the same haplotype.

**Figure 4:**
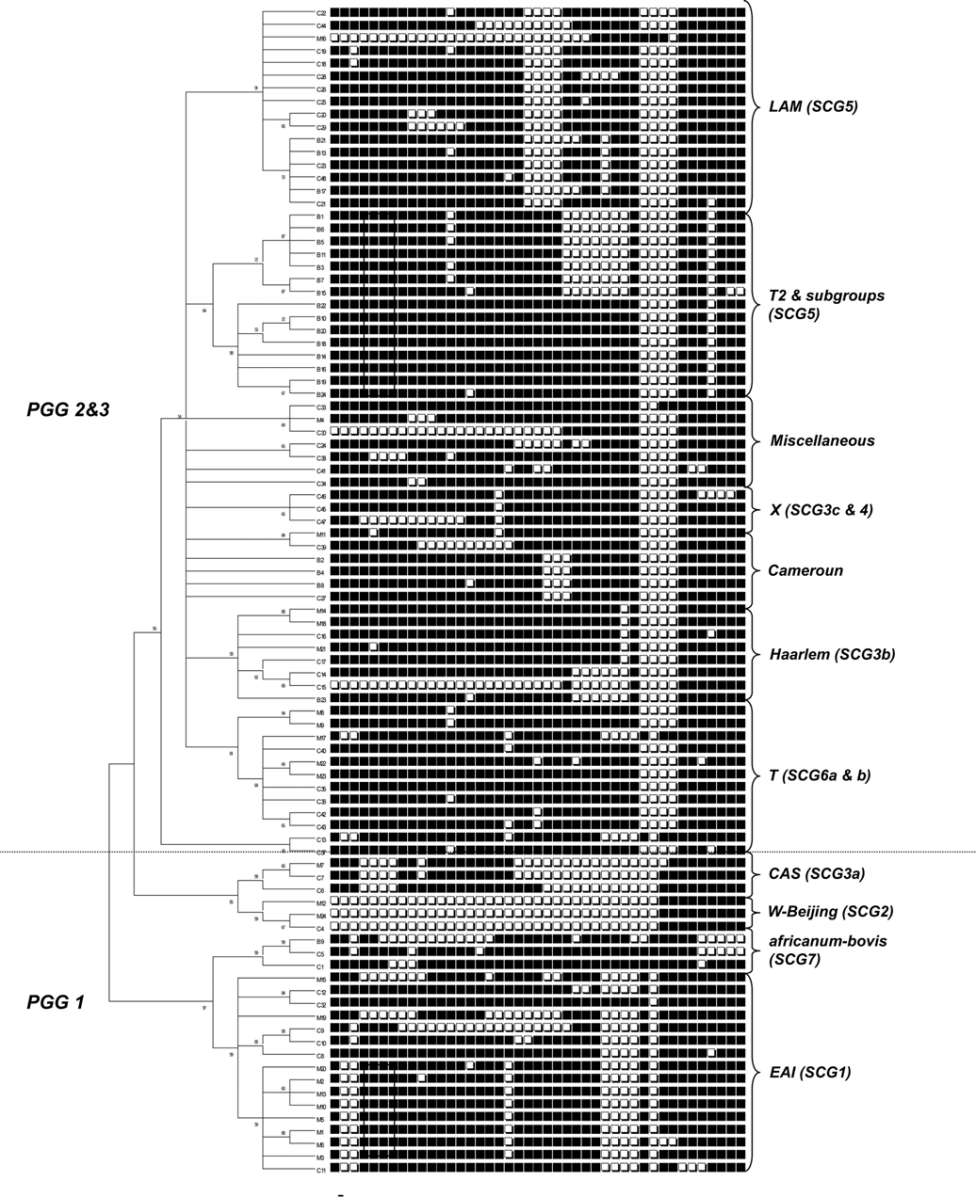
Spoligotype based unrooted tree of the strains analyzed. This unrooted neighbor-joining tree was built with the Mega software on the same dataset as in Figure 1. The upper part of the tree describes Principal Genetic Group (PGG) 2 & 3 strains and the lower part relates to PGG1. The spoligotypes are indicated next to the tree to show the excellent congruence. Clades are named according to SpolDB4 and to the recent SNP-cluster group(SCG) nomenclature.

A comprehensive set of 56 genes encoding 3R system components was analyzed by sequencing (Tables 1 and 2) in a spoligotype-based set of 92 clinical strains; 45 of these strains are representative of global MTC diversity [3] and were included to ascertain the global diversity of 3R genes in *M. tuberculosis*. The other 47 strains were chosen to allow evaluation of the resolution power of 3R-SNP-based variations in strains from very precise geographical locations. One group of strains was from Bangui, CAR, where the predominance of two major families of strains has been described: they were used to determine whether this approach could discriminate between these strains. The second group was from Madagascar, a country where both human and bacterial diversity is high. Analysis of 6.7 Mbp of MTC nucleotide sequence, corresponding to roughly 1.5 times the genome of *M. tuberculosis* H37Rv, showed an unexpectedly large set of highly polymorphic genes, implicating 3R systems in MTC evolution. We identified 259 polymorphisms, in 52 variable genes from 92 clinical isolates. These polymorphisms comprised 161 non synonymous (ns) SNPs, including three encoding stop codons, 91 synonymous (s) SNPs, and 7 deletions (Tables 1, 3, 4, 5 and 6, see Supplementary Information Text S1, Table S1). As previously reported, nsSNPs were much more abundant than sSNPs (Figure 1, Table 1) [7]. SOS repair, Holliday junction-resolving genes and NER were the classes of genes that showed nucleotide diversity lower, although similar, than that of the housekeeping genes. This surely reflects the importance of these genes for mycobacteria. It seems logical that an obligate intracellular pathogen such as *M. tuberculosis* maintains a stable SOS repair machinery and consequently stable Holliday junction-resolving genes, which are induced as part of the SOS response. NER stabillity might indicate the importance of UV radiation resistance for *M.tuberculosis*. Nevertheless, these results reveal a wealth of polymorphisms, very different from the restricted allelic variation generally observed for *M. tuberculosis* housekeeping genes (Tables 3, 4 and 5). We identified 74 haplotypes, with a nucleotide diversity per site of 0.00024, approximately twice that reported for the control group of housekeeping genes (Mann-Whitney p< = 0.01082). Due to technical limitations, the analysis of housekeeping genes was restricted to a control group of strains whose genomic sequences were available online (see Materials and Methods). In the control group, 3R and housekeeping genes showed a nucleotide diversity of 0,00042 and 0,00012, respectively, which represents a 3.5-fold difference. In total, 115 informative sites marking the evolutionary history of *M. tuberculosis* and 137 non-informative sites specific to single strains were identified. No recombination events were detected. Figures 2 and 3 show the phylogenetic networks constructed using the data obtained [15]. Polymorphic site parsimony was perfectly correlated with spoligotype signature and could therefore be used to trace the evolutionary history of MTC (Figure 4). Principal genetic group 1 (PGG1) strains, such as the W-Beijing, CAS, EAI and *M. bovis* families of strains, appear to be only very distantly related to the strains of PGG2 and PGG3, providing a strong argument for the use of ecotype categorization for MTC members rather than the traditional subspecies classification. Our results suggest a high degree of functional redundancy among 3R genes. The occurrence of an ns variation in one gene in a particular 3R system is generally accompanied by neutral mutations or wild-type copies of other genes from the same system. This may reflect the existence of an equilibrium, demonstrating the importance of these 3R systems for the genomic integrity in mycobacteria and the significance that even individual nsSNPs may have. This is demonstrated by the analysis of average nucleotide diversity per classes of genes (Figure 1), where only the groups involved in direct repair and alkylation damage, ligases and AP endonucleases show a bigger than 2-fold average non-synonymous diversity in comparison with synonymous diversity.

**Table 1:** Putative gene function and distribution of synonymous and non-synonymous SNPS, deletions and stop codons found in this study.

	sSNPs	nsSNPs	Deletions	Stop Codons	Putative Function
*ligA*	2	2	0	0	Probable DNA ligase
*ligD*	2	6	0	0	Possible ATP-dependent ligase
*ligB*	3	5	1	0	Possible ATP-dependent ligase
*ligC*	2	5	0	0	Possible ATP-dependent ligase
*ssb*	0	0	0	0	Probable single-strand binding protein
*recB*	1	5	2	0	Probable exonuclease V
*recC*	5	7	0	0	Probable exonuclease V
*recG*	1	5	0	0	Possible ATP-dependent DNA helicase
*uvrD1*	1	2	0	0	Possible ATP-dependent DNA helicase II
*uvrD2*	2	3	0	0	Possible ATP-dependent DNA helicase II
*uvrB*	3	4	0	0	Probable excinuclease ABC
*uvrC*	2	5	0	0	Probable excinuclease ABC
*uvrA*	1	3	0	0	Probable excinuclease ABC
*polA*	8	8	0	0	Probable DNA polymerase I
*ruvA*	2	0	0	0	Probable holliday junction DNA helicase
*ruvB*	3	1	0	0	Probable holliday junction DNA helicase
*ruvC*	0	0	0	0	Probable crossover junction endodeoxyribonuclease
*recA*	1	2	0	0	Recombinase A
*lexA*	0	2	0	0	Repressor lexA
*recD*	4	7	0	0	Probable exonuclease V
*recN*	3	3	0	0	Probable DNA repair protein
*mfd*	4	5	0	0	Probable transcription repair coupling factor
*nudC*	0	5	0	0	Probable NADH pyrophosphatase
*deoA*	2	3	0	0	Probable thymidine phosphohydrolase
*dnaQ*	1	6	0	0	Probable DNA polymerase III
*dnaN*	2	2	0	0	Probable DNA polymerase III
*recX*	1	1	0	0	Regulatory protein
*recO*	1	2	0	0	Possible DNA repair protein
*dut*	0	2	0	0	Probable dUTPase
*radA*	3	1	0	0	DNA repair protein
*end*	2	4	1	1	Probable endonucleaseIV
*xthA*	0	0	0	0	Probable exodeoxyribonuclease III
*recF*	0	5	0	0	DNA replication and repair protein
*tagA*	1	2	0	0	Probable DNA-3-methyladenine glycosylase I
*Rv0944*	1	3	0	0	Possible formamidopyrimidine-DNA glycosylase
*nei*	2	2	0	0	Probable endonuclease VIII
*Rv2979c*	0	3	0	0	Probable resolvase
*nth*	0	1	0	0	Probable endonuclease III
*ung*	3	1	0	0	Probable uracil-DNA glycosylase
*mutT1*	2	3	0	0	Possible hydrolase mutt1
*mutT2*	1	1	0	0	Probable 8-oxo-dGTPase
*mutT3*	1	1	0	0	Probable 8-oxo-dGTPase
*mutT4*	2	2	0	0	Probable nudix hydrolase
*ogt*	1	1	0	0	6-O-methylguanine-DNA methyltransferase
*alkA*	4	9	1	1	Probable ada regulatory protein alkA
*mpg*	0	0	0	0	Possible 3-methyladenine DNA glycosylase
*mutM*	0	2	0	0	Probable formamidopyrimide-DNA glycosylase
*mutY*	1	2	0	0	Probable adenine glycosylase
*recR*	1	1	0	0	Probable recombination protein
*dinx*	0	2	0	0	Probable dna polymerase IV
*dinF*	1	5	0	1	Possible DNA-damage-Inducible protein F
*Rv3644c*	2	3	0	0	Possible DNA polymerase
*dnaZX*	2	2	0	0	DNA polymerase III
*dinP*	3	2	0	0	Possible DNA-damage-inducible protein P
*mrr*	1	1	0	0	Probable restriction system protein
*Rv2464c*	0	1	2	0	Possible DNA glycosylase

**Table 2:** List of oligonucleotides (5′-3′) used in this study.

**ligAf**	GCGTGTGGTCTGGCCAATGCGAC	**ruvBf**	GATACGGTGCTGGCCGCCAACCAT	**mfdf**	CAATGTTGACTAACCTCGGCGCCCTAGAAT
**ligAr**	CGGTTGGGTTATCTAGCCGGCATCGT	**ruvBr**	GGGGTCATTGCCAACGGCTCCTTTG	**mfdr**	ACCGGCATTTCCTCGGTGTATTGCACCT
**ligA2**	AGGTCTTCCGGCTGGACGACTTCC	**ruvCf**	GGAAATTTACCATCGCACGTTCCATAGGCG	**mfd22**	ATTGGCTCAACGTCCACCACGGATGA
**ligA3**	TCCGAACGCGAATTCATCATGCCCA	**ruvCr**	GTGTCGCGTTCACTCGGTAGCCCACA	**mfd23**	GTGAATTCCTGGAAGCCTCGTGGTCGGT
**ligDf**	GTCACGGCGAAATTCCACGCGATATTTGA	**uvrAf**	CCTGATGGTTGTCGGTACGGGCACATAGC	**mfd24**	CGTCAGGATGGTCGACATCTCGCGAATC
**ligDr**	CCCGACCAGATCCAGCAACGACACGTC	**uvrAr**	CGTGAGTCGATGCACACCGATGAGTGAGA	**mfd25**	CAGACCGGGGTGCGCTGGAAGGA
**ligD2**	TCACCAGCGGCAGCAAGGGATTGCAT	**uvrA2**	GCTGACCGATCCGCCGAAGCTGAAA	**mfd26**	GGGATACATTCGCTTCAGCCGCACCT
**ligD3**	GATACACACCGAGGACCACCCGCTGGAATA	**uvrA3**	CGTTCCTGCAACGCAAGATGTCCCAAAC	**uvrD1f**	CCCGCAAAAACTTGGCGGGAAAAGTG
**ligBf**	CCACATAGCCCCCAGGCGGTATTGGTA	**uvrA4**	TGTCCGGCCGGGAAAGCATTGAGATAC	**uvrD1r**	GGACTTAGCGTCGGCAATTACACCGGTTGA
**ligBr**	CGCTTGGTCGACGAGCGTGAATCTG	**uvrBf**	GTGGCTCTAGGCTGGTTGGCGTGGCTT	**uvrD12**	CAACCTGAAGAACGAGTTGATCGACCC
**ligB2**	GGCACTCTACCGGGCAAAGGGTCTCAG	**uvrBr**	GAAACTCGTTGGTGGCGTAAAGCGCGAC	**uvrD13**	CGAGGGTAGCGAGATCACCTACAACGAT
**ligCf**	ACCCCAGCTTCGGGAAATACATCCTGT	**uvrB2**	GACATGTCCTTTACTCGCGGCTCGTTT	**rv3644f**	CGACGAAAGATCACGGAATTGTCGCGAA
**ligCr**	TCGCCACACAGACGACAAGTCCCAA	**uvrB3**	GGCAGACGGTGTATCTGTCTGCCAC	**rv3644r**	TCTACCGACTGAGCTAAGGCGGCTTTTCC
**ssbf**	GTAATGCGCACCGACAAGCACTAATCGG	**uvrCf**	CAATGCACCCGACCAACAGTGGGATAGC	**dnaQf**	CGGGTGGTTACCACCCGGGCAGTTTAC
**ssbr**	CCTTGTAGTCGATCGCTTGGTCCTTCTTCG	**uvrCr**	CCGGACAGCCCGGTTACCAAGACGA	**dnaQr**	TCTCGCAAGGTGTTACGGTGTTGACTGG
**recBf**	CACCCTTCGAGGTGTTGCTCGGCAA	**uvrC2**	TACATCGACAAATGTTCCGCGCCGTGT	**dnaNf**	GTCGCGGTGGGTCAGAGGTCAATGATGA
**recBr**	GTTGCGCCACATGCACATCCGACA	**uvrC3**	CGGTGCACCGAAACGCAGAAGATGC	**dnaNr**	CGCGGTGCGAACCTAACGTCGTCGAATA
**recB2**	AACTTCGGTCGTCAGGAGACCGATCCGGAG	**uvrD2f**	GGCTCGGCAGTTGTTCATGTCGCAC	**mrrf**	AGATGAGGAAGATGCGACGCCTGCAGC
**recB3**	TCAAACGGCACACGCTCGGGTATGA	**uvrD2r**	CAGCAGAATGCGACCTGGAGGTTAACCG	**mrrr**	GCACCACGACCGTGACTACAAACGAATTGC
**recB4**	GTCTTCGTGGCCAAGGGACTGCAGTTTC	**uvrD22**	GGCTGTCTACTCCGAATACGAGG	**dinFf**	CTAAGACCGTGAATTTGGCCGCGGTT
**recB5**	GTTCCGGCGCCGATCTGACATCA	**uvrD23**	GGTAGCCATTCTCTACCGAGTCAATGC	**dinFr**	GTAGCTCCGTATTCTGTCCGCCGAGTTTGC
**dnaZXf**	CGCCGAAATCACGCCGAACGTTCA	**nudCf**	AGGCCAGCGACCGGCTGCTCTATATT	**dinF2**	CGACGTTTTTGTCCTACGGCACCACAG
**dnaZXr**	CGAACGAAACAACCTGCAGCTACATCACG	**nudCr**	ACAGAACTGTTCCCACGGTGAAGTTCGC	**dinXF**	ATGGCAGCGGCTGAGGTTGATGCG
**dnaZX2**	AACACCTGATCTTCATATTCGCCACCA	**xthAf**	CTGGGCTTCCGGCTGCACCATCA	**dinXR**	CGATCCGGTGGTGATCGCGGTGTCTA
**dnaZX3**	CTGCTGCTGGAAGTGGTTTGCG	**xthAr**	GCCACGCCGCCCTGACTGAGACAA	**dinX2**	GAACAGCTTTCTTTCGATGAAGCGTTCGCC
**polAf**	AGCCCGGGCGTAAAACTGAAACGTGTTG	**endf**	CACGAATACGGACGGCAGCATCCC	**radAf**	TAATGGTGCCGATCTCGGCCGGATT
**polAr**	CGACGGGTACACGCTGGACAAACTCGGT	**endr**	CGCAAGCACAGCGGCGAGCAGTAT	**radAr**	GTTGCTGCATAGCGGACATCGAGGGAGAA
**polA2**	GTCAGCGAACTTACGCGCTTCACAC	**dinPf**	GGCGGCCATACCCTGCAAACCCT	**radA2**	GAGATCTACCTCGCCGCACAGTCCGA
**polA3**	CGAAGGCGCTTACCTCGATACCGCGACG	**dinPr**	AACGTGTTCTTCACGCGCGCGCT	**recFf**	GGAGCGAGTGTCTTTCGGGTTTACGACTGC
**polA4**	TTGTTCGACAAGACCGGGCATCCGTT	**recRf**	AAGATGGCGCAGGAACGGCTGGGT	**recFr**	CGCCCTCGACCGGCGTCTTGTCC
**recAf**	CAGCCGACTTGTCAGTGGCTGTCTCTAGTG	**recRr**	GAGATCAACATTTTGCAGGCAAGGTGCG	**mutT1f**	CAGGAATCGCTGATGGAACG
**recAr**	ATCCAATACCCGGTTGCCGATGTCTTC	**recOf**	AGAAGCGCCAACCACGGGGTACGAGA	**mutT1r**	CTCCCGCCAAGAAGGCAAC
**recA2**	ATGAGCCAGGCGCTGCGGAAAATGAC	**recOr**	TCAGGAAAGGTCGGGTAGTCGTCGGGG	**mutT2f**	CTGCCAGCCGTTGAGGTCGT
**recA3**	CTGATCGGAGATGGCAGGGATGGT	**dutf**	GAAAGCCAATGGCCACCACCACCA	**mutT2f**	CGGGCATGCAAACCCAAGTTA
**recA4**	GTCGTGGATCGCAAGCGACATATC	**dutr**	CGTCTTTGCCTGTGCGTCTACCGAATG	**mutT3f**	GTCACGTCTGTTAGGACCTC
**recCf**	TGTCGTTCGGTGATCTCGCGTCTGTTTATG	**deoAf**	CCGTCGAGGTGATCGTGCAGCAA	**mutT3r**	CGCGCAACGGCTGCCGG
**recCr**	AGACCGGCCAGCGCGAACGTCTTAC	**deoAr**	TACTGATCGACCATCCGGTGCGACC	**mutT4f**	TCGAAGGTGGGCAAATCGTG
**recC2**	CATGTCCGCCACGACAAGACCATC	**rv0944f**	AACGCACCAACTTTCTGTGACCGCGA	**mutT4r**	TGGGGTTCGCTGGAAGTGG
**recC3**	GTGGTGATGTGCCCCGACATCGACACCTAC	**rv0944r**	CTTTCAGTTCGGTCAGCCGATCCGTG	**ogtf**	CAGCGCTCGCTGGCGCC
**recC4**	CTGACCGTCTGCACGATGGTCCCGAT	**rv2464f**	ATAGTCGACGCCTTCGTCACCACG	**ogtr**	GACTCAGCCGCTCGCGA
**recC5**	AGGGGTTCTTCCGGGCGCTGGACTACA	**rv2464r**	GCCTACGTGATGAACCGCTTCGAC	**alkAf**	AGCCGCGTAGGTAACCT
**recDf**	GGTGTGTTCACCTGGAACCCGCCCA	**ungf**	GAGGAGTGGACTGGATACGCGGGCT	**alkAr**	TGCTCGAGCATCCGCAG
**recDr**	GTCGCCGTGCTGTTCGTGTATGCGATGT	**ungr**	CGGCGGCAACAAGAAGCGACTCA	**alkA2**	CGCATGCAGACCGCCCG
**recD2**	TCTCGCAAGGTGTTACGGTGTTGACTGG	**neif**	TCTGGTCGAGCGGGCCGACGGCAT	**alkA3**	CACTGCACGTTGCCGAC
**recGf**	CATGTGCACGACCACCATCCAGGCAC	**nei**	GGTGGCAGGCAATATCTGCCCAAGGCGG	**alkA4**	GCTGACGATGCCGTTGCC
**recGr**	CGATGATCCCAGCGTCTGATACGCGA	**nthf**	ATGACACAAGGAGAGTAAACATGGC	**mutMf**	CTGGTTCGATGGTGATGACC
**recG2**	CAGCACAAAAGTGCAGAGCTGGGACATCTT	**nthr**	AATAGTCATGCAGTTGGGCAACCA	**mutMr**	GTGCGCTCGACCCACAG
**recG3**	GATGACGGCAGGGCAGAAGAAGCAAGTTC	**rv2979f**	GTTCGAAGGTCCACAGGGCCAGAACG	**mutYf**	CCGGCGACGAATCGCTCGTT
**recNf**	TGTGTCACGTCCGTCAAATGGGCAC	**rv2979r**	TCCAGTTGTATGCCTTGCGACGAGCA	**mutYr**	AGCTGGGACAGTCGTCGCGG
**recNr**	GGTGAAGCTGGGCAGGTGCGTGGA	**mpgf**	TTTACCACGGATGACGCGCAGCTGGT		
**recN2**	AAGCTGCGGGATGCCTGGCTAACGG	**mpgr**	GGATCGAAGCCGGCGTACACCGTCAT		
**recXf**	CCGACGTGGCTGACGAGATCGAGAAGAA	**tagAf**	TGAGCTCGAGGCGCTACGCTCTCAGC		
**recXr**	CCGCCATCAAGTCGAGGTAAATTCGTTCA	**tagAr**	CCCCGCCATTGGATTTCCAGCCATA		
**ruvAf**	TTTGGCGCTGGCGATCTGTCACTGTTG	**lexAf**	CGAATGCGACTACATTCATTGCCATGAAC		
**ruvAr**	GCTGGCCGATGAATTCGCGTAACGAG	**lexAr**	CGAACTCTGGTTCGCCAGTGAAGAAGTG		

The name of the target gene and position of the oligonucleotide is followed by the oligonucleotide sequence. (f) for forward and (r) for reverse oligonucleotides used for amplification and sequencing reactions. Oligonucleotides whose name finishes in number were used for sequencing reactions.

**Table 3:** DNA polymorphism and divergence data.

Gene	*N° Haplotypes*	*Nucleotide diversity*	*k*	*Pi (a)/Pi (s)*	*Ka/Ks ratio*
***recB***	7	0,00014	0,462	7,406	31,414
***dnaQ***	8	0,00094	0,934	2,780	10,491
***uvrC***	8	0,00021	0,399	0,731	6,132
***dinF***	7	0,00022	0,290	5,225	5,497
***alkA***	14	0,00066	0,991	1,781	4,115
***ogt***	3	0,00037	0,182	2,577	2,794
***ligD***	8	0,00028	0,638	0,424	1,981
***mfd***	9	0,00022	0,818	0,346	1,389
***recX***	3	0,00016	0,086	1,130	1,156
***deoA***	6	0,00012	0,151	0,935	0,948
***uvrB***	7	0,00010	0,216	0,840	0,839
***end***	5	0,00028	0,215	0,809	0,811
***mutT2***	3	0,00015	0,065	0,755	0,763
***uvrD1***	4	0,00003	0,065	0,712	0,712
***tagA***	4	0,00011	0,065	0,696	0,696
***uvrA***	5	0,00004	0,108	0,556	0,550
***polA***	12	0,00052	1,412	0,464	0,495
***recN***	7	0,00026	0,461	0,068	0,463
***mutT4***	4	0,00017	0,129	0,380	0,386
***uvrD2***	5	0,00006	0,130	0,383	0,381
***dinP***	6	0,00012	0,130	0,373	0,376
***Rv0944***	4	0,00027	0,129	0,380	0,371
***ligA***	5	0,00004	0,087	0,364	0,364
***mrr***	3	0,00005	0,043	0,352	0,352
***recA***	4	0,00004	0,086	0,350	0,346
***ung***	5	0,00059	0,407	0,242	0,247
***dnaZX***	5	0,00014	0,252	0,201	0,195
***mutT3***	3	0,00010	0,065	0,189	0,187
***ruvB***	4	0,00008	0,086	0,131	0,130
***ligB***	9	0,00030	0,464	0,151	0,129
***mutY***	4	0,00018	0,167	0,134	0,127
***recC***	13	0,00031	1,005	0,397	0,115
***mutT1***	6	0,00030	0,290	0,104	0,097
***recR***	3	0,00056	0,340	0,086	0,075
***ligC***	8	0,00040	0,435	1,324	0,059
***recD***	11	0,00042	0,731	0,195	0,051
***recG***	7	0,00018	0,399	0,139	0,023
***dnaN***	5	0,00035	0,418	0,071	0,015
***Rv3644c***	5	0,00057	0,689	0,076	0,013
***nei***	5	0,00061	0,465	0,039	0,011
***recO***	4	0,00042	0,334	0,061	0,010
***radA***	5	0,00025	0,356	0,026	0,005
***ruvA***	3	0,00011	0,065	0,000	0,000
***mutM***	3	0,00005	0,043	------	------
***Rv2464c***	2	0,00008	0,064	------	------
***nth***	2	0,00009	0,064	------	------
***lexA***	3	0,00010	0,065	------	------
***recF***	6	0,00013	0,151	------	------
***dinX***	3	0,00022	0,312	------	------
***dut***	3	0,00044	0,204	------	------
***nudC***	6	0,00049	0,460	------	------
***Rv2979c***	3	0,00083	0,486	------	------
***total***	**74**	**0,00024**	**17,109**	**0,451**	**0,384**

The genes for which no Pi(a)/Pi(s) and Ka/Ks ratios could be determined are marked by -----.

**Table 4:** DNA polymorphism data on the control group of strains.

*3R genes*	*Nucleotide diversity*	*k*
***ligA***	0,00014	0,286
***ligD***	0,00050	1,143
***ligB***	0,00075	1,143
***ligC***	0,00027	0,286
***recB***	0,00070	2,286
***recC***	0,00066	2,190
***recG***	0,00000	0,000
***uvrD1***	0,00012	0,286
***uvrD2***	0,00000	0,000
***uvrB***	0,00050	1,048
***uvrC***	0,00015	0,286
***uvrA***	0,00029	0,857
***polA***	0,00032	0,857
***ruvA***	0,00000	0,000
***ruvB***	0,00000	0,000
***recA***	0,00060	1,429
***lexA***	0,00044	0,286
***recD***	0,00033	0,571
***recN***	0,00065	1,143
***mfd***	0,00039	1,429
***nudC***	0,00121	1,143
***deoA***	0,00089	1,143
***dnaQ***	0,00144	1,429
***dnaN***	0,00071	0,857
***recX***	0,00000	0,000
***recO***	0,00000	0,000
***dut***	0,00184	0,857
***radA***	0,00000	0,000
***end***	0,00038	0,286
***recF***	0,00000	0,000
***tagA***	0,00000	0,000
***Rv0944***	0,00240	1,143
***nei***	0,00112	0,857
***Rv2979c***	0,00195	1,143
***nth***	0,00039	0,286
***ung***	0,00042	0,286
***mutT1***	0,00000	0,000
***mutT2***	0,00000	0,000
***mutT3***	0,00000	0,000
***mutT4***	0,00076	0,571
***ogt***	0,00057	0,286
***alkA***	0,00070	1,048
***mutM***	0,00033	0,286
***mutY***	0,00031	0,286
***recR***	0,00000	0,000
***dinx***	0,00021	0,286
***dinF***	0,00087	1,143
***Rv3644c***	0,00024	0,286
***dnaZX***	0,00000	0,000
***dinP***	0,00000	0,000
***mrr***	0,00031	0,286
***Rv2464c***	0,00000	0,000
**total**	**0,00042**	**29,714**

The 3R genes were analyzed from the strains *M. bovis subsp. bovis* AF2122/97 and *M. tuberculosis* CDC1551 from the TIGR website at http://cmr.tigr.org, *M. microti* and *M. africanum* from the Sanger Institute at http://www.sanger.ac.uk and strains F11, C and *Haarlem* from Broad Institute available at http://www.broad.mit.edu.

**Table 5:** DNA polymorphism data on the control group of strains.

*Housekeeping genes (hsk)*	*Nucleotide diversity*	*k*
***rplM***	0,00050	0,222
***rplA***	0,00031	0,222
***rplB***	0,00026	0,222
***rplC***	0,00000	0,000
***rplD***	0,00000	0,000
***rplE***	0,00000	0,000
***rplF***	0,00000	0,000
***rplJ***	0,00000	0,000
***rplN***	0,00000	0,000
***rplP***	0,00000	0,000
***total***	***0,00012***	***0,667***

The housekeeping genes were analyzed from the strains *M. bovis subsp. bovis* AF2122/97 and *M. tuberculosis* CDC1551 from the TIGR website at http://cmr.tigr.org, *M. microti* and *M. africanum* from the Sanger Institute at http://www.sanger.ac.uk and strains F11, C and *Haarlem* from Broad Institute available at http://www.broad.mit.edu.

**Table 6:** Outcome of correlating the location of non-synonymous single nucleotide polymorphisms (ns SNPs) inside genes, the amino acids they are predicted to encode and predicted enzymatic signature motifs and active sites.

**Genes with ns SNPs predicted as non-significant (amino acid changes can possibly only indirectly induce steric changes)**
*recA, recB, recC, recD, recG, recN, recR recX, ligA, ligB, ligC ligD, lexA, uvrA, uvrB, uvrC, mutY, nth, nei, fpg, mfd, ung, mutT1, mutT2, mutT4-Rv3908, Rv3644c, dnaZX, mrr, Rv2464c, dut, radA, Rv0944, Rv2979c, nudC, deoA, dnaO, dinF, dinP, dinX, dnaN, ruvB*
**Genes with ns SNPs in 3R genes predicted to be significant (encoding amino acid changes located in predicted enzymatic signature motifs and active sites**
*alkA, dinP, polA, ligC, recO, tagA, mutT2, dinX*
**Genes displaying no sequence variation among 100 MTC strains**
*ssb*, *xthA*, *mpg* and *ruvC*

### Major 3R findings

We investigated the location of ns SNPs and compared the amino acids they encoded with predicted enzymatic signature motifs and active sites. Significant polymorphisms in 3R genes were observed for particular MTC families, that may be progenitors for altered mutator phenotypes (see SI Text S1 for further information about the genes studied, the SNPs found and inferences about their significance). For example, one W-Beijing strain shows an accumulation of ns variations in the *tagA* and *alkA* genes. The *tagA* gene encodes a 3-methyladenine DNA glycosylase I, is constitutively expressed and highly specific, whereas *alkA* encodes a 3-methyladenine DNA glycosylase II—an alkylation damage-inducible protein capable of catalyzing the excision of a wide variety of alkylated bases. The *tagA* gene was one of the most conserved genes in our panel, with ns variants found in only two strains. The observation of such variants in only one of the W-Beijing strains is most interesting, and consistent with recent observations that the pathogenic characteristics of W-Beijing strains are not conserved, with strains within individual W-Beijing lineages having evolved unique pathogenic characteristics [16]. Another case concerns *M. bovis* strains, which displayed the greatest accumulation of mutations in *recBCD* genes. RecBCD processes DNA ends resulting from double strand breaks, acting as a bipolar helicase that splits the duplex into its component strands and digests them until a recombinational hot-spot (chi site) is encountered. This association is of interest because the formation of deletions has been identified as a common feature for RecB mutants, including in *M. bovis* strains [2]. In addition, the gene encoding the recombination factor RecO had ns SNPs predicted to cause amino acid substitutions affecting component locations critical to enzymatic function. Furthermore, two SNPs were found in the gene encoding the DNA glycosylase End (codon-167 coupled with a codon-170 Gly-Ser variation) and a combination of two ns variations was found in the gene encoding the DNA polymerase PolA (codon-186 and codon-188) . However, only the ns SNPs in the *polA* gene were at locations that could affect active sites in the expression product. MTC strains are highly clonal, so the occurrence of two variations in the same codon coupled with another variation only two codons away seems unlikely to be either random or entirely fortuitous; rather is indicative of strong natural selection either seperately or due to epistasis. However, this does not exclude a possible recombination or horizontal transfer event. Some of the most polymorphic genes, such as those encoding components of the RecBCD pathway, made it possible to distinguish 24 haplotypes among the strains analyzed. RecB, RecC and RecD orthologs have a limited species distribution, being found in only a few enterobacteria in addition to *M. tuberculosis* and *B. burgdorferi*. This has led to the suggestion that *M. tuberculosis* acquired these genes through a lateral transfer event [17]. The highly polymorphic genes also included *polA*, dinP, dinX and *dnaQ*, which displayed a remarkable accumulation of ns variations, suggestive of changes in PolIII proofreading in the strains possessing them. Furthermore, significant ns SNPs were detected in the genes encoding LigC and MutT2, and MutT2 has already been suggested to be involved as a source of variation in w-Beijing strains (13). Other molecules potentially affected by SNP-encoded amino-acid substitutions, albeit to a lesser extent, included DNA polymerases DinP and DinX, the recombination factors RecB, RecC, RecD and RecN, the ligases LigA, LigB, LigC and LigD, the nucleotide excision repair [18] components UvrA, UvrB, and UvrC. The nsSNPs in the genes encoding the BER DNA glycosylases MutY, Nth, Nei and Fpg, recombination proteins RecA, RecC, RecG, RecN and RecR, LexA, the NER components Uvr, UvrC and the helicase UvrD, and the double-stranded DNA translocase and ATPase RuvB led to predicted amino-acid changes at component sites which could potentially induce steric changes only indirectly (Table 6).

Although the 3R genes were unexpectedly polymorphic, these results are fully consistent with the idea that *M. tuberculosis* is a strictly clonal organism, and provide no evidence of recent lateral gene transfer [19]. It has previously been suggested that the occurrence of amino-acid substitutions in *M. tuberculosis* strains is strongly indicative of possible functional consequences of these substitutions [8]. This might induce slack in the fidelity of genome maintenance and could be regarded as compensation for the genetic isolation of MTC strains, devoid as they are of horizontal gene transfer. In addition to the lack of a recognizable mismatch repair system, the predicted reduced stringency/precision in DNA repair resulting from the polymorphisms detected, might facilitate or even allow adaptation. However, this does not necessarily mean that the selective consequences of non synonymous changes are immediately effective [20].

### Effect on evolution

**Figure 5:**
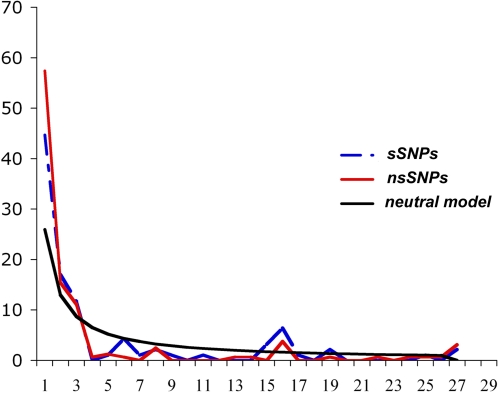
Site frequency spectrum of sSNPs and nsSNPs. This spectrum summarizes the allele frequencies of the various mutations in the sample.

We investigated whether some form of natural selection could account for the patterns of diversity observed, by comparing the site frequency spectrum (SFS) of synonymous and non synonymous variants (Figure 5). The frequency spectrum is a count of the number of mutations that exist at a frequency of xi = i/n for i = 1, 2,…, n−1, in a sample of size n. In other words, it represents a summary of the allele frequencies of the various mutations in the sample. In a standard neutral model (i.e., a model with random mating, constant population size, no population subdivision, etc.), the expected value of xi is proportional to 1/i. Selection against deleterious mutations will increase the fraction of mutations segregating at low frequencies in the sample. A selective sweep has roughly the same effect on the frequency spectrum. Conversely, positive selection will tend to increase the frequency in a sample of mutations segregating at high frequencies. Under a strictly neutral model, these two classes of genetic variants should present a similar SFS [21]. The higher values observed for the singleton ns SNPs than for s SNPs are suggestive of negative/purifying selection. However, caution is required in interpretation, because different selective/demographic scenarios may mimic similar patterns of diversity. Negative selection alone and/or population growth might be equally likely to account for the patterns observed [21]. We compared the non synonymous and synonymous substitution rates for each gene, by calculating the ratio of non synonymous mutations per non synonymous site (*Ka*) to synonymous mutations per synonymous site (*Ks*) (Tables 3, 4 and 5). Under a strictly neutral model of evolution, this ratio should be equal to one. For this particular analysis, we used the oldest strain from the panel analyzed (as determined by the number of spoligotype spacers) as the outgroup. Nine of the 52 genes presented K_A_/K_S_ values higher than 1. Six of these nine genes had considerably higher K_A_/K_S _ratios, suggesting that the evolution of these genes might have been driven by positive natural selection. The remaining genes had K_A_/K_S_ ratios below one, consistent with negative/purifying selection, as suggested by the SFS spectrum. Further detailed evolutionary studies will be required to elucidate the evolutionary forces that may account for the patterns observed, and to determine which of these genes have contributed significantly to the evolution of *M. tuberculosis*.

### New phylogenetic tool

*M. tuberculosis* strains are highly clonal. However, SNP analysis of 3R genes seems to be a robust phylogenetic method with very high resolution, even for a generally monomorphic, recent pathogen, such as *M. tuberculosis*. Genome stability is a key factor in maintenance of the integrity of an organism. Nevertheless, genome variability may sometimes be a selective advantage. Pathogenic bacteria are constantly exposed to hostile conditions, in which factors such as host defenses and antibiotic treatments are continuously changing their environments. Provided that it is in balance with bacterial fitness, a mutator phenotype may act as a driving force facilitating strain evolution, through, for example, the acquisition of antibiotic resistance, virulence factor variation and adaptation to the genetic stress conditions exerted by the environment (e.g. host defense mechanisms). Changes in mutation rates generally result from allelic variation in the genes controlling 3R fidelity [22], [23]. The 3R polymorphisms observed in this study suggest that these genes in general may be subjected to negative/purifying selection pressure. In this model, a large number of the variations observed would be expected to be deleterious, at least to some extent. We consistently found 3R polymorphisms to be frequent in a global panel of *M. tuberculosis* families, indicating that most of these mutations can be only slightly deleterious, as fitness costs would otherwise be too high for these MTC strains to sustain with such a wide range of human hosts; highly deleterious mutations would be expected to give rise to non-viable cells and would therefore be selected against. These classes of “slightly deleterious” mutations may also result in suboptimal 3R system activity. Deficiencies in polymerase proofreading activity, for example, might cause an increase in mutation rates, whereas incorrect non-homologous end joining might result in deletions or other polymorphisms. These events could potentially increase genomic variability and might therefore be a selective advantage to the strains possessing them under certain stressful conditions, whereas selection against them would be expected in changing environments [22]. Overall, this study shows that 3R gene family polymorphisms can be used to study the evolution of highly clonal bacteria, and in particular MTC strains. It also provides a powerful new high-resolution tool for strain discrimination for clinicians. The high-resolution surveillance of haplotypes with particular characteristics could be used to provide early warning of the spread of localized epidemics, making it easier to deal with outbreaks caused by MDR and XDR MTC strains, for example, and facilitating their dissemination.

## Materials and Methods

DNA was sequenced directly, with fragments amplified by the dideoxy chain-termination method from the strains described above. In the comparison of the nucleotide diversity of 3R and housekeeping genes in the control group of strains, the analysis of housekeeping genes was restricted to a control group of strains whose genomic sequences were available online: *M. bovis subsp. bovis* AF2122/97 and *M. tuberculosis* CDC1551 strains from the TIGR website at http://cmr.tigr.org, *M. microti* and *M. africanum* strains from the Sanger Institute at http://www.sanger.ac.uk and strains F11, C and *Haarlem* from Broad Institute available at http://www.broad.mit.edu. The sSNPs and nsSNPs were concatenated, resulting in a single character string (nucleotide sequence) for each clinical isolate analyzed. Network software [15] was initially used for phylogenetic and molecular evolution analysis. This software assumes that there is no recombination between genomes. Phylogenetic trees were built with the neighbor-joining method and MEGA software [24]. The DNAsp package [25] was used to analyze the average nucleotide diversity of the MTC and interspecies Ka/Ks tests. Prediction of the 3R protein secondary structure was performed based on sequence alignments with various 3R homologs by using the JPred program. A search for functional domains or signatures in the 3R gene deduced amino acid sequences was carried out using the DOLOP, the PROSITE and the Pfam databases. The presence of recognized DNA binding motifs and active sites was assessed by using the Expasy site and PFAM bioinformatics algorithms available at http://us.expasy.org/cgi-bin/protscale.pl, and the electrostatic charge was calculated by using the EMBOSS package (http://proteas.uio.no/EMBOSS). Thereby, the significance of nsSNPs in relation to predicted DNA binding and enzymatic signature motifs and active sites was predicted.

## Supporting Information

Text S1 Supporting information about the genes studied, the SNPs found and inferences about their significance.
(0.24 MB DOC)

Table S1 Full results from this study. The First line, in red, represents the strains to which the results refer. A denomination starting with (B) means that the strains belong to the Bangui CAR group, conversingly an (M) and a (C) indicates that the strains belong to the Madagascar or Global groups, respectively. The first column indicates the gene where the mutation is present. The second column indicates the genomic position where the polymorphism was found. Polymorphism are marked in red. Non-synonymous polymorphism are indicated by a red genomic position.
(0.48 MB XLS)
