# Supplementary material for: Evolution and Diversity of Clonal Bacteria: The Paradigm of Mycobacterium tuberculosis
Source: PLoS One. 2008 Feb 6;3(2):e1538. doi: 10.1371/journal.pone.0001538 (PMC2211405; doi:10.1371/journal.pone.0001538)
Supplement: Text S1 — Supporting information about the genes studied, the SNPs found and inferences about their significance. (0.24 MB DOC) [file pone.0001538.s001.doc]

# Supplementary information for

**Evolution and Diversity of Highly Clonal Bacteria; the Paradigm of *Mycobacterium tuberculosis***

Tiago Dos Vultos, Olga Mestre, Jean Rauzier, Marcin Golec, Nalin Rastogi, Voahangy Rasolofo, Tone Tonjum, Christophe Sola, Ivan Matic, Brigitte Gicquel †

† To whom correspondence should be addressed. E-mail: bgicquel@pasteur.fr

**This file includes:**

Supplementary Information Text

***Polymorphisms of 3R family genes***

We carried out single nucleotide polymorphism (SNP) analysis on a panel of *Mycobacterium tuberculosis* complex (MTC) strains from around the world. *M. tuberculosis* has homologs of key DNA repair proteins present in *Escherichia coli* for all the principal, traditional DNA repair pathways. The DNA repair, replication and recombination (3R) genes described are depicted as they appear, as homologs of *Escherichia coli* genes, with the corresponding annotated functions. MTC genomes have revealed no genes encoding recognized DNA mismatch repair proteins. Nevertheless, the characterization of *M. tuberculosis* DNA repair components is still in its infancy and based principally on sequence homology searches. The existence of currently unknown mechanisms of DNA mismatch repair cannot be excluded.

Among the 56 genes, analyzed in this study, encoding 3R functions, *ssb*, *xthA*, *mpg* and *ruvC* were the only genes displaying no variation. The *ssb* gene encodes a probable single-strand-binding protein, the role of which is to protect transiently formed ssDNA from nuclease and chemical attack, and to prevent it from forming aberrant secondary structures. This gene has thus been included in a set of 262 genes thought to be the minimal requirement for cellular life [1,2]. The *xthA* gene encodes a protein involved in the base excision pathway of DNA repair in bacteria. Its product has endonucleolytic activity, cleaving the 5' phosphodiester bond adjacent to spontaneous or induced abasic sites in DNA [3]. Mutants defective in this enzyme are hypersensitive to UV radiation and hydrogen peroxide [4-6]. *Mpg*, a gene with no homolog in *E. coli*, encodes a 3-methylpurine-DNA glycosylase that initiates DNA base excision repair and has a wide substrate range, including damaged or altered bases in duplex DNA [7]. *RuvC* encodes a dimeric endonuclease that resolves the Holliday junction into duplex products by introducing symmetric nicks in two of the four DNA strands [8,9]. This gene, unlike its *E*. *coli* counterpart, is induced by DNA damage in *M. tuberculosis*. It has been shown to be important for *Helicobacter pylori* survival in macrophages, suggesting its possible importance for other pathogens, including *M. tuberculosis* [10,11].

***Nucleotide variations in base excision repair (BER) genes***

One of the most frequently occurring stable and abundant oxidized base lesions in DNA is 8-oxo-7,8-dihydroguanine (8oxoG). This lesion has strong promutagenic properties [12] and, during replication, 8oxoG frequently mispairs with the nucleotide A [13], leading to G:C → T:A transversions [14]. Another important oxidized purine lesion is formamidopyrimidine (faPy), which has mostly cytotoxic effects [15]. Subtle base changes in DNA, such as these, are normally repaired by the base excision repair (BER) pathway [16]. BER is initiated by DNA glycosylases, which cleave the *N*-glycosylic bond and remove the damaged base. This event is followed by strand cleavage of the sugar-phosphate backbone, either by the AP-lyase activity inherent to many DNA glycosylases or by an AP endonuclease. The repair process is completed by the successive actions of phosphonucleotide kinase or a 3’- or 5’-deoxyribosephosphodiesterase, a DNA polymerase and a DNA ligase [16,17].

In *E. coli*, alkylated bases are repaired by two DNA glycosylases, products of the *tagA* and *alkA* genes. The *tagA* gene encodes 3-methyladenine DNA glycosylase I and is constitutively expressed; this enzyme is highly specific [18]. *AlkA* encodes 3-methyladenine DNA glycosylase II, an alkylation damage-inducible protein that catalyzes the excision of a wide variety of alkylated bases, due to its versatile active site. This enzyme is a component of the adaptive response controlled by *ada* [19]. The *ada* gene encodes O^6^-alkylguanine-DNA alkyltransferase I, which prevents transition mutations by repairing O-alkylation damage to guanine and thymine, acts as sensor for alkylation damage in DNA, activates the transcription of genes encoding products required for the adaptive response and is involved in the termination of this kind of response [20]. A second type of O^6^-alkylguanine-DNA alkyltransferase is encoded by the *ogt* gene in *E. coli*. In *M. tuberculosis*, *ada* and *alkA* are predicted to encode fused proteins and to be part of an operon including *ogt* [21]. The *ada/alkA* were found to be highly polymorphic, with a large number of the strains analyzed showing mutations. The variants include an 79 AMBER codon in strains from the Central African Republic and a codon-12 Ile-Val substitution in strains of PGG1, in the *ada* part of the operon, suggesting that the induction of both *alkA* and *ogt* might be altered in these variants [20]. The only non synonymous *ogt* variation (nsSNPs) found, a codon-15 Thr to Ser substitution characteristic of the Haarlem genotype, was coupled with wild-type *ada/alkA,* in contrast to the synonymous variation (sSNP) found in strains with nsSNP variations in the *ada* part of this gene. This pattern of variation is of interest because it might serve as the basis for the dichotomy between selective pressures on an active gene and neutral variations in a supposedly inactive gene. The *tagA* gene is well conserved, with the exception of a codon-129 Ala-Thr substitution found in a W-Beijing family strain and a codon-170 Tyr-Ser variation found in one *M. africanum* strain. This may indicate a compensatory function for the other highly polymorphic 3-methyladenine-DNA glycosylase or may simply reflect the greater risk of damage from 3-methyladenine and 3-ethyladenine than from other agents when AlkA is active.

Uracil residues can be introduced into genomic DNA by polymerases using dUTP rather than dTTP or by deamination of the existing dCMP residues [22-24]. The amount of dUTP in the nucleotide pool is controlled in *E. coli* by a dUTP pyrophosphatase encoded by the *dut* gene [20]. Mutations in this gene were either lethal or increased the incorporation of uracil residues into genomic DNA *[25]*. The uracil-DNA glycosylase Ung removes this base from the DNA [26]. Ung is important for prevention of mutations, for increasing resistance to reactive nitrogen intermediates generated by acidified nitrite, and for the multiplication of GC-rich bacteria in macrophages (in the *M. smegmatis* and *P. aeruginosa* models) [27]. One fifth of the strains analyzed possessed an nsSNP in either *dut* or *ung*. There was also a degree of complementation between these two genes, because an nsSNP in one gene was, in all cases, associated with a wild-type sequence or an sSNP in the other, suggesting that the absence of both genes may be detrimental. Furthermore, the Haarlem genotype codon122 Val-Leu substitution in *dut* was always linked to a synonymous codon-167 variation in *ung*. These two genes encode functionally related proteins, so it is unclear whether the sSNP observed is merely a result of neutral evolution or an evolutionary scar of a previous altered state, due, for example, to the reversion of a damaging nsSNP. The *deoA* gene encodes a thymidine phosphorylase that facilitates the use of exogenous thymidine. Some mutations have been found to allow the more efficient conversion of exogenous thymidine to dTTP, which can then compete with dUTP for incorporation into DNA [28]. However, our study revealed no non synonymous variations in *deoA* in strains of the Haarlem genotype.

In *E. coli*, the repair of 8oxoG and faPy lesions is initiated by the activity of a DNA glycosylase of the formamidopyrimidine-DNA glycosylase (Fpg) / endonuclease VIII (Nei) family. *E. coli* Fpg, also known as MutM, catalyzes the excision of 8oxoG and other purines with oxidative damage from the DNA, whereas the principal substrates of *E. coli* Nei are oxidized pyrimidines. Fpg and Nei have different substrate specificities, but have common N- and C-terminal domains and a similar enzymatic mode of action, with three types of activity: hydrolysis of the *N*-glycosylic bond with the transient formation of an AP site (DNA glycosylase activity) and cleavage of the sugar-phosphate backbone 3’ to the abasic site (ß-elimination) before 5'-cleavage (d-elimination) [29-31]. The sequential performance of these functions removes the lesion from duplex DNA in which a single-nucleotide gap in the damaged strand is left flanked by phosphate residues. Nei displays DNA glycosylase/AP lyase activity and excises modified pyrimidines, including thymine glycol, uracil glycol, dihydrothymine, dihydrouracil, 5-hydroxycytosine, 5-hydroxyuracil, and β-ureidoisobutyric acid [31]. Nei and MutM differ in structure from the members of the larger Nth family of DNA glycosylases, but a Nei homolog nonetheless serves as a backup for Nth activity in knockout mice [32]. Furthermore, under some conditions, such as the repair of X-ray- or hydrogen peroxide-induced lethal lesions, Nei and Nth can substitute for each other [33]. The endonuclease III Nth displays some sequence similarity to MutY, but has a very different substrate specificity and is involved in the removal of a wide range of thymine residues damaged by ring saturation, fragmentation, or ring contraction. Like Nei, it has AP lyase activity [34]. Nth, together with Nei, probably forms part of the repair system dealing with 8-oxoG. 8-oxoG residues incorporated opposite G are removed by the Nth protein and Nei excises 8-oxoG paired with an A or G [35,36]. Only two strains displayed nsSNPs in the *nei* gene: one carried a codon-206 Trp-Cys substitution and the other, a 235-stop codon that presumably abolishes expression. Only W-Beijing strains carried variations of the *nth* gene, with a codon-2 Pro-Arg substitution.

In addition to Fpg/Nei, the DNA glycosylase MutY and the 8oxodGTPase MutT are involved in handling the potential mutagenic effects of guanine oxidation. MutY removes adenine mispaired with 8oxoG, whereas MutT scavenges the nucleotide pool for oxidized dGTPs. This triplet of DNA repair enzymes comprises the G^O^ system [37]. *M. tuberculosis* harbors no less than four copies of the *fpg*/*nei* genes, and four MutT homologs have been annotated in mycobacterial genomes, which contain only a single *mutY* homolog [38,39]. Nudix hydrolases are enzymes catalyzing reactions in which the substrate is a nucleoside diphosphate attached to some other moiety **X** [40] [41]. Oxidized guanine is a potent mutagen because of its ambiguous pairing with cytosine and adenine. The *E. coli* MutT nudix hydrolase protein specifically hydrolyzes both 8-oxo-deoxyguanosine triphosphate (8-oxo-dGTP) and 8-oxo-guanosine triphosphate (8-oxo-rGTP), preventing their misincorporation into DNA and RNA opposite template A [41-45]. When nsSNPs were located in the genes encoding the Go components MutY, Fpg and MutT, the predicted amino acid changes were mostly located in component sites at which they would not induce steric changes. Our analysis of the four putative *mutT* genes, *mutY* and three of the *mutM* homologs — *fpg, Rv2464c* and *Rv0944* — thus suggests that these genes are important and that the maintenance and stability of this ubiquitous system of DNA repair may be essential. We identified nsSNPs in all the genes, but there seemed to be a highly conserved set of genes in which the previously observed equilibrium was consistently maintained: an nsSNP in one of these genes was accompanied in most cases by wild-type sequences or sSNPs in the others. However, exceptions were observed: in the ancestral *EAI1* clade, codon-102 Ile-Leu and codon-236 Ala-Asp variations were observed within the *mutY* and *mutM* genes, respectively, and in the *M. africanum* strains, codon-67 Asn-Asp and codon-14 Arg-Trp variations were found in *mutM* and *mutT1*.

Nei, MutM and Nth have some properties in common. This led us to compare their polymorphisms. As in other groups of enzymes, the presence of nsSNPs in one gene was associated with no significant variation in the other. *M. tuberculosis* AlkA and Nei double mutants were particularly sensitive to nitrous acid; however, we found no coupled nsSNPs for these two genes [46].

Most of the DNA glycosylases considered above recognize and remove damaged or inappropriate bases from the genomic DNA by cleaving the N-glycosyl bond, thereby giving rise to AP sites. These sites can also arise spontaneously in DNA. They are noncoding during semiconservative DNA synthesis, and most replicative polymerases are strongly inhibited by an AP site in the template DNA [20]. These lesions are repaired by AP endonucleases, including XthA an invariable gene in our study (see above). However a second gene, encoding the endonuclease IV End (Nfo) may also fulfill this function. Both exonuclease III and endonuclease IV cleave DNA 5’ to an AP site, exonuclease III being the more active of the two [47]. A single nucleotide deletion (SND) was found in codon-172 of the *end* gene in several strains, whereas the T3 Osaka strain contains a stop mutation at codon-233; this enzyme therefore cannot be active in these strains. Two SNPs were also found in codon-167 of *end* in two T3 strains, together with a codon-170 Gly-Ser substitution. Mycobacteria are highly clonal, and the spontaneous occurrence of two variants at the same codon, coupled with another only three codons away is highly unlikely. These findings therefore suggest that a recombination event may have occurred. Collectively, all these results strongly confirm that the product of the *xthA* gene may be functionally more important than endonuclease IV/Nfo.

***Nucleotide variations in DNA polymerase genes***

AP endonuclease activity always occurs 5’ to the AP site, leaving 3’-OH and 5’-deoxyribosephosphate (dRp) termini. In the BER pathway, the removal of this 5’-dRp residue is followed by gap filling, mediated principally by the polymerase I, PolA [48]. The *M. tuberculosis* *polA* gene was one of the genes with the largest number of variants among the genes included in our study. W-Beijing genotype strains present a combination of two ns variations, at codon-186 and codon-188, raising questions about the integrity of this polymerase function; as for *end*, this is suggestive of recombination. However, this is not an isolated case, because some EAI strains also display two SNPs at codon-660, suggesting that *polA* may be particularly prone to recombination. All the polymerase genes analyzed were highly polymorphic. *Rv3644c*, and *dnaZX*, *dnaQ* and *dnaN*, encoding the gamma/tau, epsilon and beta subunits of polymerase III, had levels of variability similar to that of *polA*. Nevertheless, many of these variations were silent, with the exception of those in the epsilon subunit of pol III, which is generally thought to be involved in proofreading. The *dnaQ* gene in PGG1 strains carried up to three coupled nsSNPs, suggesting alterations to the proofreading activity of pol III in these strains.

*M. tuberculosis* contains two apparently functionally redundant replicative DNA polymerases: DnaE1 and DnaE2. Error-prone DNA repair being the major function of one of these enzymes would provide one possible explanation for this redundancy. In *M. tuberculosis,* the DNA polymerase DnaE2 has been shown to be upregulated by UV-induced DNA damage and to be a major mediator of induced mutagenesis in mice, playing a role in the emergence of drug resistance [49]. These data suggest that DnaE2, and not a member of the Y family of error-prone DNA polymerases, is the primary mediator of survival through inducible mutagenesis. As the gene encoding DnaE2 was not included in our original gene panel, we have assessed only potential SNPs emerging from existing MTC genome sequences and identified on the basis of gene alignments. The alignment of CDC1551, F11, 210, *africanum*, *microti* and *bovis* *dnaE2* sequences available at the [www.tigr.org](http://www.tigr.org/), [www.sanger.ac.uk](http://www.sanger.ac.uk/) and [www.broad.mit.edu](http://www.broad.mit.edu/) revealed no non synonymous polymorphism with respect to H37Rv. These findings illustrate the potential importance of this gene in mycobacteria.

***Nucleotide variation in DNA ligase genes***

The nicks resulting from excision repair or from the action of another damaging agent may be joined by the formation of a phosphodiester bond. The enzymes catalyzing this reaction are called DNA ligases. This reaction requires energy, which may be provided by either ATP or NAD^+^. Most eubacteria, like *E. coli*, have DNA ligases that use NAD^+^ as their source of energy. *E. coli* carries a single DNA ligase, which uses NAD^+^ as a nucleotide cofactor and is encoded by the *ligA* gene. Remarkably, *M. tuberculosis* has one NAD^+^-dependent DNA ligase gene, *ligA*, and three distinct ATP-dependent DNA ligase gene homologs, *ligB*, *ligC* and *Rv0938* (*ligD*). LigA may be essential for repair and recombination, and therefore for viability, in *M. tuberculosis* [50] . In contrast, the ATP-dependent DNA ligases were highly polymorphic. Most significantly, SNDs were observed in codon-179 of *ligB* in strains of the Cameroon genotype and a LigD codon-656 ns variation was found to be coupled with *ligC* codon-176 and *ligB* codon-224 nsSNPs in the EAI1 and EAI3 strains, respectively. *M. tuberculosis* H37Rv, considered to be a recent member of the MTC, has nsSNPs in all three ATP-dependent DNA ligases. This high level of polymorphism probably reflects functional redundancy among these three DNA ligases, or may be due to neutral evolution. As for the groups of enzymes described above, balanced polymorphisms are observed, probably allowing the bacteria to maintain essential functions.

***Nucleotide variations in nucleotide excision repair [24] genes***

Nucleotide excision repair [24] is an alternative pathway to BER, in which genomic damage is repaired by means of incisions in regions flanking the damaged DNA, leading to the excision of an oligonucleotide rather than a single base. This system can recognize a wider range of damaged bases than the BER system, due to the use of more generic endonucleases. This pathway involves the three components of the excinuclease ABC — UvrA, UvrB and Uvr — the DNA helicase II UvrD and the transcription repair-coupling factor (TRCF), encoded by the *mfd* gene. *UvrA* is a member of the group of SOS genes, the transcription of which is induced by agents causing certain types of damage. UvrA recognizes helix-distorting DNA damage by binding as a dimer to the altered DNA, leading to the formation of a (UvrA)_2_UvrB DNA complex. UvrA then dissociates from the complex, leaving a stable UvrB-DNA complex. UvrB has a high affinity for UvrC, which is repositioned so that it can initiate the catalysis of incisions 5’ and 3’ to the DNA damage. The release of the oligonucleotide defined by these two incisions and the turnover of the bound UvrB and UvrC proteins are catalyzed by the DNA helicase II, UvrD, and PolA. Following incision, DNA helicase II (UvrD) releases the UvrC protein and the damaged oligonucleotide, leaving a UvrB-gapped DNA complex. PolA then binds to the 3’OH terminus generated at the 5’ incision and displaces the bound UvrB protein during gapped DNA synthesis. TRCF is required for NER activity upon transcriptional arrest due to base damage in the template DNA strands [20]. *UvrA* was the least polymorphic of the genes encoding components of the ABC excinuclease. *UvrB* and *UvrC* showed similar levels of variability. Double codon-289 and -434 *uvrC* non synonymous variations were observed in W-Beijing genotype strains, and codon-32 and -460 *uvrB* nsSNPs in the *M. africanum* strain. Nevertheless, the most interesting observation in terms of NER components was that none of the strains studied carried variations in more than one of these three genes. Our findings indicate that UvrA damage recognition activity is highly important, as the corresponding gene displayed the lowest level of variation, and also that the functions of UvrB and UvrC may be redundant. Surprisingly, although most bacteria carry just one gene encoding DNA helicase II, *M. tuberculosis* possesses two putative genes encoding this protein: *uvrD1* and *uvrD2*. This suggests that the products of these two genes may play an important role in stabilizing the genome of *M. tuberculosis,* as they were conserved in the strains studied. Only two strains had nsSNPs in *uvrD1* and three strains had nsSNPs in *uvrD2*. This may reflect the relative importance of NER in mycobacteria. A significant number of strains harbored an *mfd* gene with non synonymous mutations. This may be a sign of a deficiency in dealing with transcriptional arrest by base damage in template DNA, or may simply indicate that this kind of arrest is rare in *M. tuberculosis.*

*M. tuberculosis* also has a DNA damage induction pathway independent of classical LexA/RecA regulation [51] and non-homologous end-joining [52], both of which have not been identified in *E. coli*. The effect of these processes on mycobacterial genome stability, survival and latency is currently unclear and requires further examination.

***Nucleotide variation in recombination (Rec) genes***

Two major RecA-dependent pathways are involved in initiating homologous recombination: the RecBCD and RecFOR pathways. RecBCD is a multifunctional enzyme complex that processes DNA ends resulting from a double-strand break. RecBCD is a bipolar helicase that splits the duplex into its component strands and digests them until it reaches a recombinational hotspot (chi site), whereas RecFOR is important in post-replication daughter-strand gap repair [53,54]. Both pathways provide an ssDNA molecule coated with RecA, facilitating the invasion of a homologous molecule. Strand exchange is then promoted by RecA. The resulting joint molecules formed by RecA can then be resolved either by the RuvABC complex or by the action of the recG helicase. Deletions are a commonly reported feature of RecB mutants. Interestingly, the *M. bovis* strains analyzed all had an SND at codon-860 in *recB*. This observation, together with the large number of regions of difference (Rds) characterizing *M. bovis* strains [55], suggests that RecB was involved in the emergence of the *M. bovis* genotype. The genes encoding the components of the RecFOR pathway seem to be more strongly conserved than those encoding the components of the RecBCD pathway. RecR interacts with both RecO and RecF, but no three-component complex has ever been described [53,56]. RecR may therefore play a central role in this pathway. Most Central African Republic Strains had an sSNP in this gene, whereas all the W-Beijing genotype strains had a wild-type sequence. Curiously, only nsSNPs were found in the *recF* gene, in only a small number of strains. This may indicate the action of recent selection pressure on this gene. The same applies to the three RecFOR components, because most of the nsSNPs appeared as singletons in these genes. RecBCD component-encoding genes were highly polymorphic among MTC members. Nevertheless the occurrence of several nsSNPs in more than one RecBCD component was rare. An nsSNP in one component of the RecBCD or RecFOR pathway was generally accompanied by wild-type or synonymous variations in the components of the other pathway, suggesting interplay between the Rec components; the strains of the W-Beijing genotype provided an exception, with RuvC invariable (see above), and RuvAB components also highly conserved, only one strain having an nsSNP in these genes. EAI strains carried an sSNP in the *recG* gene, which had only singleton nsSNPs. This strong conservation seems to confirm the importance of the RuvABC complex and RecG, consistent with their almost ubiquitous presence in bacteria.

Other proteins reported to be important for recombinational repair were studied and found to be highly conserved. These proteins included RecN, which appears to promote faithful double-strand break repair [57], and the RecA-related recombinase RadA.

*M. tuberculosis* has the genes required for a functional SOS system, but not *polB* and *umuD*. The RuvABC complex, consisting of the products of the *recN*, *uvrA*, *uvrB*, and *uvrD* genes, is discussed above. RecA and LexA play an important role in regulating this system, and *dinP* and *dinX*, which are presumed to encode PolV and PolIV, respectively, are involved in mutagenesis. Variations in these genes, with the exception of a frequent codon-306 variation common to LAM genotype strains, were mostly restricted to single strains.

**Location of nsSNPs with respect to signature motifs**

We assessed the location of nsSNPs and the amino-acids encoded with respect to enzymatic signature motifs and active sites, and found that only in AlkA, PolA and the recombination factors RecB, RecC and RecG were the nsSNPs predicted to cause amino acid substitutions affecting component locations critical to enzymatic function. Other components potentially affected by the amino-acid substitutions induced by SNPs, although to a lesser extent, included the DNA polymerases DinP and DinF, the recombination factors RecD and RecN, the ligases LigA, LigB, LigC and LigD, the nucleotide excision repair [24] components UvrA, UvrB, and UvrC and the regulatory component Mfd. In contrast, nsSNPs in the genes encoding the BER DNA glycosylases MutY, Nth and Fpg, RecA and LexA, the NER helicase UvrD, and the double-stranded DNA translocase and ATPase RuvB, resulted in predicted amino acid changes at sites with no steric effects.

**MATERIALS AND METHODS**

**Clinical MTC isolates and genes**

We selected 92 DNAs from three collections: a panel of 43 DNAs corresponding to MTC strains representing worldwide, spoligotype-based, genetic diversity [58], and two collections of 23 strains from Bangui, Central African Republic [59] and24 strains from Madagascar. These last two collections were included to assess whether there were major variations that might account for the selective advantages of certain strain families in geographically restricted areas. The 23 DNAs from Bangui and the Central African Republic were selected at random and had no apparent epidemiological links. They belonged to a group of MDR strains including a family of strains prone to MDR acquisition. The 24 strains from Madagascar were also MDR strains selected at random, with no apparent epidemiological links, and were chosen to represent the rich demographic history of the population of this island and the expected diversity of strains infecting patients in this country.

**Sequencing primers and methods**

The full list of genes assessed and the corresponding list of oligonucleotides used in this study is given in Table S2. DNA was sequenced directly, with fragments amplified by the dideoxy chain-termination method, using the Big Dye Terminator Cycle Sequencing Kit (Perkin Elmer Applied Biosystems, Courtaboeuf, France) on a GeneAmp polymerase chain reaction (PCR) system 9600 (Perkin Elmer). The sequencing products were run on a DNA analysis system model 373 or 3100 (Applied Biosystems). Reference sequences of *M. tuberculosis* H37rv genes were obtained from the Institut Pasteur website at <http://genolist.pasteur.fr/TubercuList/>

**Data analysis, phylogenetic methods, population genetics methods**

The sSNPs and nsSNPs were concatenated, resulting in a single character string (nucleotide sequence) for each clinical isolate analyzed. Network software [60] was initially used for phylogenetic and molecular evolution analysis. This software assumes that there is no recombination between genomes. DNAsp software [61] was also used to analyze the genetic diversity and population genetics of the MTC. Phylogenetic trees were built with the neighbor-joining method and MEGA software [62]. Deletions and mutation 5, which may be homoplasic, were not used in the construction of phylogenetic trees. Due to the presence of e nucleotide deletion sites in certain clades, the pairwise deletion option was used to process gaps/missing data. Default values (including the Kimura 2 parameter model) were otherwise used. The EXPASY and PFAM bioinformatics algorithms were used to assess the significance of nsSNPs.

**BIBLIOGRAPHY**

1. Mushegian AR, Koonin EV (1996) A minimal gene set for cellular life derived by comparison of complete bacterial genomes. Proc Natl Acad Sci U S A 93: 10268-10273.

2. Saikrishnan K, Jeyakanthan J, Venkatesh J, Acharya N, Sekar K, et al. (2003) Structure of Mycobacterium tuberculosis single-stranded DNA-binding protein. Variability in quaternary structure and its implications. J Mol Biol 331: 385-393.

3. White BJ, Hochhauser SJ, Cintron NM, Weiss B (1976) Genetic mapping of xthA, the structural gene for exonuclease III in Escherichia coli K-12. J Bacteriol 126: 1082-1088.

4. Demple B, Halbrook J, Linn S (1983) Escherichia coli xth mutants are hypersensitive to hydrogen peroxide. J Bacteriol 153: 1079-1082.

5. Sammartano LJ, Tuveson RW (1983) Escherichia coli xthA mutants are sensitive to inactivation by broad-spectrum near-UV (300- to 400-nm) radiation. J Bacteriol 156: 904-906.

6. Souza LL, Eduardo IR, Padula M, Leitao AC (2006) Endonuclease IV and exonuclease III are involved in the repair and mutagenesis of DNA lesions induced by UVB in Escherichia coli. Mutagenesis 21: 125-130.

7. Biswas T, Clos LJ, 2nd, SantaLucia J, Jr., Mitra S, Roy R (2002) Binding of specific DNA base-pair mismatches by N-methylpurine-DNA glycosylase and its implication in initial damage recognition. J Mol Biol 320: 503-513.

8. Iwasaki H, Takahagi M, Shiba T, Nakata A, Shinagawa H (1991) Escherichia coli RuvC protein is an endonuclease that resolves the Holliday structure. Embo J 10: 4381-4389.

9. Sharples GJ, Ingleston SM, Lloyd RG (1999) Holliday junction processing in bacteria: insights from the evolutionary conservation of RuvABC, RecG, and RusA. J Bacteriol 181: 5543-5550.

10. Brooks PC, Movahedzadeh F, Davis EO (2001) Identification of some DNA damage-inducible genes of Mycobacterium tuberculosis: apparent lack of correlation with LexA binding. J Bacteriol 183: 4459-4467.

11. Loughlin MF, Barnard FM, Jenkins D, Sharples GJ, Jenks PJ (2003) Helicobacter pylori mutants defective in RuvC Holliday junction resolvase display reduced macrophage survival and spontaneous clearance from the murine gastric mucosa. Infect Immun 71: 2022-2031.

12. Demple B, Harrison L (1994) Repair of oxidative damage to DNA: enzymology and biology. Annu Rev Biochem 63: 915-948.

13. Shibutani S, Takeshita M, Grollman AP (1991) Insertion of specific bases during DNA synthesis past the oxidation-damaged base 8-oxodG. Nature 349: 431-434.

14. Cheng KC, Cahill DS, Kasai H, Nishimura S, Loeb LA (1992) 8-Hydroxyguanine, an abundant form of oxidative DNA damage, causes G----T and A----C substitutions. J Biol Chem 267: 166-172.

15. Boiteux S, Laval J (1983) Imidazole open ring 7-methylguanine: an inhibitor of DNA synthesis. Biochem Biophys Res Commun 110: 552-558.

16. Seeberg E, Eide L, Bjoras M (1995) The base excision repair pathway. Trends Biochem Sci 20: 391-397.

17. Slupphaug G, Kavli B, Krokan HE (2003) The interacting pathways for prevention and repair of oxidative DNA damage. Mutat Res 531: 231-251.

18. Bjelland S, Bjoras M, Seeberg E (1993) Excision of 3-methylguanine from alkylated DNA by 3-methyladenine DNA glycosylase I of Escherichia coli. Nucleic Acids Res 21: 2045-2049.

19. O'Brien PJ, Ellenberger T (2004) The Escherichia coli 3-methyladenine DNA glycosylase AlkA has a remarkably versatile active site. J Biol Chem 279: 26876-26884.

20. Friedberg EC, Walker GC, Siede W (1995) DNA repair and mutagenesis. Washington, D.C.: ASM. xvii,698p.,[698]p. of plates p.

21. Durbach SI, Springer B, Machowski EE, North RJ, Papavinasasundaram KG, et al. (2003) DNA alkylation damage as a sensor of nitrosative stress in Mycobacterium tuberculosis. Infect Immun 71: 997-1000.

22. Duncan BK, Weiss B (1982) Specific mutator effects of ung (uracil-DNA glycosylase) mutations in Escherichia coli. J Bacteriol 151: 750-755.

23. Mosbaugh DW (1988) Purification and characterization of porcine liver DNA polymerase gamma: utilization of dUTP and dTTP during in vitro DNA synthesis. Nucleic Acids Res 16: 5645-5659.

24. Tye BK, Chien J, Lehman IR, Duncan BK, Warner HR (1978) Uracil incorporation: a source of pulse-labeled DNA fragments in the replication of the Escherichia coli chromosome. Proc Natl Acad Sci U S A 75: 233-237.

25. Wang L, Weiss B (1992) dcd (dCTP deaminase) gene of Escherichia coli: mapping, cloning, sequencing, and identification as a locus of suppressors of lethal dut (dUTPase) mutations. J Bacteriol 174: 5647-5653.

26. Lindahl T (1974) An N-glycosidase from Escherichia coli that releases free uracil from DNA containing deaminated cytosine residues. Proc Natl Acad Sci U S A 71: 3649-3653.

27. Venkatesh J, Kumar P, Krishna PS, Manjunath R, Varshney U (2003) Importance of uracil DNA glycosylase in Pseudomonas aeruginosa and Mycobacterium smegmatis, G+C-rich bacteria, in mutation prevention, tolerance to acidified nitrite, and endurance in mouse macrophages. J Biol Chem 278: 24350-24358.

28. el-Hajj HH, Zhang H, Weiss B (1988) Lethality of a dut (deoxyuridine triphosphatase) mutation in Escherichia coli. J Bacteriol 170: 1069-1075.

29. Bhagwat M, Gerlt JA (1996) 3'- and 5'-strand cleavage reactions catalyzed by the Fpg protein from Escherichia coli occur via successive beta- and delta-elimination mechanisms, respectively. Biochemistry 35: 659-665.

30. Melamede RJ, Hatahet Z, Kow YW, Ide H, Wallace SS (1994) Isolation and characterization of endonuclease VIII from Escherichia coli. Biochemistry 33: 1255-1264.

31. Zharkov DO, Golan G, Gilboa R, Fernandes AS, Gerchman SE, et al. (2002) Structural analysis of an Escherichia coli endonuclease VIII covalent reaction intermediate. Embo J 21: 789-800.

32. Takao M, Kanno S, Kobayashi K, Zhang QM, Yonei S, et al. (2002) A back-up glycosylase in Nth1 knock-out mice is a functional Nei (endonuclease VIII) homologue. J Biol Chem 277: 42205-42213.

33. Jiang D, Hatahet Z, Blaisdell JO, Melamede RJ, Wallace SS (1997) Escherichia coli endonuclease VIII: cloning, sequencing, and overexpression of the nei structural gene and characterization of nei and nei nth mutants. J Bacteriol 179: 3773-3782.

34. Breimer LH, Lindahl T (1984) DNA glycosylase activities for thymine residues damaged by ring saturation, fragmentation, or ring contraction are functions of endonuclease III in Escherichia coli. J Biol Chem 259: 5543-5548.

35. Hazra TK, Izumi T, Venkataraman R, Kow YW, Dizdaroglu M, et al. (2000) Characterization of a novel 8-oxoguanine-DNA glycosylase activity in Escherichia coli and identification of the enzyme as endonuclease VIII. J Biol Chem 275: 27762-27767.

36. Matsumoto Y, Zhang QM, Takao M, Yasui A, Yonei S (2001) Escherichia coli Nth and human hNTH1 DNA glycosylases are involved in removal of 8-oxoguanine from 8-oxoguanine/guanine mispairs in DNA. Nucleic Acids Res 29: 1975-1981.

37. Michaels ML, Cruz C, Grollman AP, Miller JH (1992) Evidence that MutY and MutM combine to prevent mutations by an oxidatively damaged form of guanine in DNA. Proc Natl Acad Sci U S A 89: 7022-7025.

38. Cole ST, Brosch R, Parkhill J, Garnier T, Churcher C, et al. (1998) Deciphering the biology of Mycobacterium tuberculosis from the complete genome sequence. Nature 393: 537-544.

39. Mizrahi V, Andersen SJ (1998) DNA repair in Mycobacterium tuberculosis. What have we learnt from the genome sequence? Mol Microbiol 29: 1331-1339.

40. Bessman MJ, Frick DN, O'Handley SF (1996) The MutT proteins or "Nudix" hydrolases, a family of versatile, widely distributed, "housecleaning" enzymes. J Biol Chem 271: 25059-25062.

41. Tajiri T, Maki H, Sekiguchi M (1995) Functional cooperation of MutT, MutM and MutY proteins in preventing mutations caused by spontaneous oxidation of guanine nucleotide in Escherichia coli. Mutat Res 336: 257-267.

42. Bhatnagar SK, Bullions LC, Bessman MJ (1991) Characterization of the mutT nucleoside triphosphatase of Escherichia coli. J Biol Chem 266: 9050-9054.

43. Ito R, Hayakawa H, Sekiguchi M, Ishibashi T (2005) Multiple enzyme activities of Escherichia coli MutT protein for sanitization of DNA and RNA precursor pools. Biochemistry 44: 6670-6674.

44. Cabrera M, Nghiem Y, Miller JH (1988) mutM, a second mutator locus in Escherichia coli that generates G.C----T.A transversions. J Bacteriol 170: 5405-5407.

45. Sekiguchi M, Tsuzuki T (2002) Oxidative nucleotide damage: consequences and prevention. Oncogene 21: 8895-8904.

46. Terato H, Masaoka A, Asagoshi K, Honsho A, Ohyama Y, et al. (2002) Novel repair activities of AlkA (3-methyladenine DNA glycosylase II) and endonuclease VIII for xanthine and oxanine, guanine lesions induced by nitric oxide and nitrous acid. Nucleic Acids Res 30: 4975-4984.

47. Cunningham RP, Saporito SM, Spitzer SG, Weiss B (1986) Endonuclease IV (nfo) mutant of Escherichia coli. J Bacteriol 168: 1120-1127.

48. Dianov G, Lindahl T (1994) Reconstitution of the DNA base excision-repair pathway. Curr Biol 4: 1069-1076.

49. Boshoff HI, Reed MB, Barry CE, 3rd, Mizrahi V (2003) DnaE2 polymerase contributes to in vivo survival and the emergence of drug resistance in Mycobacterium tuberculosis. Cell 113: 183-193.

50. Gong C, Martins A, Bongiorno P, Glickman M, Shuman S (2004) Biochemical and genetic analysis of the four DNA ligases of mycobacteria. J Biol Chem 279: 20594-20606.

51. Davis EO, Springer B, Gopaul KK, Papavinasasundaram KG, Sander P, et al. (2002) DNA damage induction of recA in Mycobacterium tuberculosis independently of RecA and LexA. Mol Microbiol 46: 791-800.

52. Della M, Palmbos PL, Tseng HM, Tonkin LM, Daley JM, et al. (2004) Mycobacterial Ku and ligase proteins constitute a two-component NHEJ repair machine. Science 306: 683-685.

53. Morimatsu K, Kowalczykowski SC (2003) RecFOR proteins load RecA protein onto gapped DNA to accelerate DNA strand exchange: a universal step of recombinational repair. Mol Cell 11: 1337-1347.

54. Singleton MR, Dillingham MS, Gaudier M, Kowalczykowski SC, Wigley DB (2004) Crystal structure of RecBCD enzyme reveals a machine for processing DNA breaks. Nature 432: 187-193.

55. Brosch R, Gordon SV, Marmiesse M, Brodin P, Buchrieser C, et al. (2002) A new evolutionary scenario for the Mycobacterium tuberculosis complex. Proc Natl Acad Sci U S A 99: 3684-3689.

56. Webb BL, Cox MM, Inman RB (1997) Recombinational DNA repair: the RecF and RecR proteins limit the extension of RecA filaments beyond single-strand DNA gaps. Cell 91: 347-356.

57. Meddows TR, Savory AP, Grove JI, Moore T, Lloyd RG (2005) RecN protein and transcription factor DksA combine to promote faithful recombinational repair of DNA double-strand breaks. Mol Microbiol 57: 97-110.

58. Brudey K, Driscoll JR, Rigouts L, Prodinger WM, Gori A, et al. (2006) Mycobacterium tuberculosis complex genetic diversity: mining the fourth international spoligotyping database (SpolDB4) for classification, population genetics and epidemiology. BMC Microbiol 6: 23.

59. Nouvel LX, Kassa-Kelembho E, Dos Vultos T, Zandanga G, Rauzier J, et al. (2006) Multidrug-resistant Mycobacterium tuberculosis, Bangui, Central African Republic. Emerg Infect Dis 12: 1454-1456.

60. Bandelt HJ, Forster P, Rohl A (1999) Median-joining networks for inferring intraspecific phylogenies. Mol Biol Evol 16: 37-48.

61. Rozas J, Sanchez-DelBarrio JC, Messeguer X, Rozas R (2003) DnaSP, DNA polymorphism analyses by the coalescent and other methods. Bioinformatics 19: 2496-2497.

62. Kumar S, Tamura K, Nei M (2004) MEGA3: Integrated software for Molecular Evolutionary Genetics Analysis and sequence alignment. Brief Bioinform 5: 150-163.

63. Filliol I, Motiwala AS, Cavatore M, Qi W, Hazbon MH, et al. (2006) Global phylogeny of Mycobacterium tuberculosis based on single nucleotide polymorphism (SNP) analysis: insights into tuberculosis evolution, phylogenetic accuracy of other DNA fingerprinting systems, and recommendations for a minimal standard SNP set. J Bacteriol 188: 759-772.
